# The First Percutaneous Closures of Patent Ductus Arteriosus in Premature Neonates in Serbia: A Case Report Series

**DOI:** 10.3390/reports8020097

**Published:** 2025-06-18

**Authors:** Stasa Krasic, Branislav Mojsic, Vladislav Vukomanovic

**Affiliations:** 1 Cardiology Department, Mother and Child Health Institute of Serbia, 11070 Belgrade, Serbia; 2 Faculty of Medicine, University of Belgrade, 11000 Belgrade, Serbia; 3 Anesthesiology Department, Mother and Child Health Institute of Serbia, 11070 Belgrade, Serbia

**Keywords:** persistent ductus arteriosus, preterm newborn, transcatheter closure, Amplatzer Piccolo Occluder

## Abstract

**Background and Clinical Significance:** The incidence of persistent ductus arteriosus (PDA) in preterm infants is the highest and depends on their birth weight (BW) and respiratory condition after birth. Previously, after the unsuccessful drug treatment, surgical ligation was the primary treatment option. However, according to clinical studies, the Amplatzer Piccolo Occluder was approved for PDA closure for patients ≥700 g. In our country, percutaneous PDA embolization has not been performed yet. **Case Presentation:** We present three premature infants with hemodynamically significant patent ductus arteriosus (hsPDA) in whom percutaneous occlusion was performed using the Amplatzer Piccolo Occluder (APO). The average gestational week (GW) was 27 ± 1, while body weight was 1030 ± 60 g. All patients had respiratory deterioration, with dilatation of the left heart chambers, and renal failure. The second developed a severe form of broncho-pulmonary dysplasia. Transthoracic echocardiography (TTE) examinations revealed a hemodynamically significant PDA (LA/Ao 1.8–2.2) and medical closure was unsuccessfully carried out. Due to the hemodynamically significant PDA maintenance in all neonates, transvenous PDA closure was performed using the APO (APO 9-PDAP-04-02-L, 9-PDAP-04-04-L, 9-PDAP-05-054L, respectively). The entire devices, with both retention discs, are implanted within the duct. TTE pointed out adequate device position without descending aorta, left pulmonary artery obstruction, residual shunt, and reverse remodelling of the left ventricle and left atrium. The first newborn was weaned from mechanical ventilation three days after the procedure and discharged three weeks after. The second patient was extubated 2 weeks after the procedure, and even the severe BPD, X-ray showed improvement. The third patient’s renal failure completely resolved, weaned from inotropic drug support and mechanical ventilation. **Conclusions**: Due to a significantly lower complication rate than surgical ligation, we will strive to make percutaneous PDA occlusion a new standard for treatment in newborns, especially preterm newborns, in our country.

## 1. Introduction and Clinical Significance

In fetal life, the ductus arteriosus (DA) connects the main pulmonary artery to the descending aorta and diverts oxygenated blood from the placenta to bypass the uninflated fetal lungs and enter the systemic circulation [1,2]. Low oxygen tension, prostanoids (mainly prostaglandin E2 (PGE2) and prostacyclin (PGI2)), adenosine, atrial natriuretic peptide, carbon monoxide, and potassium channels handle fetal DA patency. In preterm infants’ hypoxia, high pulmonary pressure, fewer layers of contractile smooth muscle cells, deficiency of vasa vasorum, and increased sensitivity to the vasodilating impact of PGE2 and nitric oxide (NO), coupled with downregulation of the receptors, lead to persistent DA (PDA) [1]. Consequently, the incidence of PDA in preterm infants is the highest and depends on their birth weight (BW) and respiratory condition after birth [1].

The clinical outcomes depend on the extent of left-to-right shunting and the steal phenomenon, which can result in reduced blood flow to the brain, kidneys, and intestines. Additionally, an increase in pulmonary blood flow, combined with low plasma oncotic pressure and heightened capillary permeability in preterm infants, may lead to hemorrhagic pulmonary edema, decreased lung compliance, and a deterioration in respiratory status. This progression can ultimately result in chronic lung disease. Furthermore, the left-to-right shunt may cause volume overload in the left ventricle and lead to congestive heart failure. These issues arise from the over-circulation of the pulmonary vascular bed and a chronic hyperdynamic state. Thus, the hemodynamically significant patent ductus arteriosus (hsPDA) leads to clinical deterioration in the respiratory and cardiovascular status of preterm infants by increasing the need for ventilator support and contributing to the development of congestive heart failure [1,2].

Medical management aimed at closing the hsPDA typically consists of intravenous administration of cyclooxygenase inhibitors or acetaminophen, both of which are utilized off-label [2,3]. Conversely, the impact of NSAIDs on prostaglandins and the reduction in blood flow to the intestine can lead to the development of intestinal perforations. Furthermore, a case of gastric bleeding was documented, resulting in the discontinuation of ibuprofen treatment. Additionally, transient renal failure was reported in 11% of patients undergoing ibuprofen therapy [4].

Ligation should be reserved only for infants who failed pharmacologic treatment and continue to have the hsPDA. This procedure usually uses an open thoracic approach, where either a metal clip is applied or the vessel is tied off. However, PDA ligation comes with several potential adverse effects, including vocal cord paralysis, postoperative hypotension, accidental ligation of bronchus, pulmonary artery or descending aorta, diaphragm paralysis, chylothorax, bronchopulmonary dysplasia, and impaired neurodevelopment [3,4,5,6]. Until now, if the hsPDA persists beyond 2 weeks of age despite pharmacologic therapy of up to 2 courses, our patients have undergone surgical ligation.

Intravascular procedures involving the placement of occluding devices were previously only available for patients weighing more than 5 kg. However, recent clinical evidence suggests that transcatheter closure (TCC) of PDA can be performed safely and effectively in premature infants with very low birth weight (VLBW) and extremely low birth weight (ELBW). Currently, percutaneous embolization of patent ductus arteriosus (PDA) is an established method for closing the condition in infants [5,6]. However, this technique has not yet been performed in our country or in other countries in the region.

We presented two preterm newborns born at 29 and 26 gestational weeks (GW), in whom PDA were percutaneously closed after unsuccessful medical treatment.

## 2. Case Presentation

### 2.1. Case 1

The second child from the twin pregnancy, which was complicated by the mother’s hypertension and premature rupture of membranes (PROM) in the first twin, was born via emergency cesarian section at 29.6 GW (BW 1370 g, BL 39 cm, AS 2/5). Corticosteroid therapy was applied antenatally to mature the lungs of the fetus. At delivery, the newborn was hypotonic, without spontaneous respirations, and bradycardic. Consequently, he was intubated, and high-frequency oscillatory ventilation was initiated. The surfactant was administered endotracheally twice. Initial antimicrobial therapy (ampicillin, amikacin) and caffeine-citrate were applied. On the second day of life (DOL), the general condition worsened with an acute pulmonary hemorrhage, so epinephrine was administered endotracheally, and ventilation parameters were corrected to a maximum FiO_2_ of 100%. Meropenem was prescribed, along with fluconazole prophylactically. On the second, fourth, and eighth DOL, convulsions were registered, so he was treated with phenobarbitone. Transthoracic echocardiography (TTE) examinations revealed a hsPDA, and medical closure was started using acetaminophen with inotropic stimulation with dopamine. After three days of drug administration, the TTE finding was maintained; the administration of paracetamol continued with inotropic stimulation with dopamine and dobutamine, but without success. Conventional mechanical ventilation is carried out from the eighth day with corticosteroid therapy according to the DART (Dexamethasone: A Randomized Trial) protocol.

**Figure 1:**
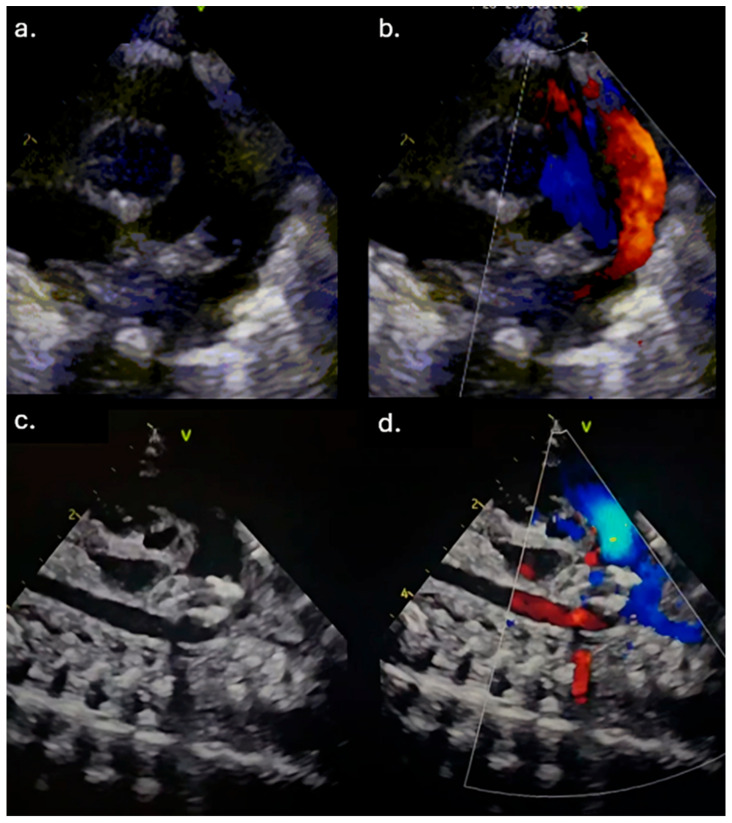
(**a**) Echocardiography examination before patent ductus arteriosus (PDA) closure. (**b**) Moderate left-to-right shunt across PDA. (**c**,**d**) Echocardiography pointed out the intraductal position of the Amplatzer Piccolo Occluder.

Due to the maintenance of the hsPDA and in addition to the applied therapy, a transfer to our Institute for further treatment was indicated. Upon admission, the patient was hypotrophic (weight 1170 g), on mechanical ventilation, normotensive, but oliguric, with signs of acute renal impairment manifested by elevated urea and creatinine levels (maximum up to 18.4 mmol/L and 90 μmol/L (ref. range 3.3–7.5 mmol/L and <55 μmol/L, respectively). TTE examination revealed a widely open PDA, 2.3 mm in diameter on the pulmonary end (2.1 mm/kg) and 8 mm long (Figure 1a,b). The left atrial to the aortic root (LA/Ao) ratio was 1.8, and the end-diastolic left ventricle diameter Z score was +2.89. The reversal of forward flow in the renal arteries during diastole was observed. Although the failed response to medical treatment was observed, the case was discussed in a joint cardiology–cardiothoracic (JCC) meeting, and we decided to perform transcatheter PDA closure using the Amplatzer Piccolo Occluder (APO) (Abbott Structural Heart, Plymouth, MN, USA) from the pulmonary arterial end.

### 2.2. Case 2

The second child from the second pregnancy experienced complications due to the mother’s hypertension and vaginal bleeding at 21 weeks gestation. The mother was treated with nifedipine, euthyrox, progesterone, and dexamethasone. The vaginal delivery was in 26 GW (BW 850 g, AS 7). Initially, the patient was treated in a regional hospital. She twice received surfactant endotracheal, but she was dependent on mechanical ventilation with high tidal volume for over a month. Consequently, the patient developed broncho-pulmonary dysplasia. Initial antimicrobial therapy (ampicillin, amikacin) and caffeine-citrate were applied. During the in-hospital stay, the patient developed septic shock and was treated with human intravenous immunoglobulins with inotropic drug support (dopamine and dobutamine), diuretics, and hydrocortisone. Due to hsPDA, paracetamol and ibuprofen were administered. However, in addition to the applied therapy, the hsPDA was maintained, so a transfer to our Institute for further treatment was indicated.

Upon admission, the patient was hypotrophic (weight 1340 g), on mechanical ventilation, and normotensive. TTE examination revealed a widely open PDA, 2.6 mm in diameter on the pulmonary end (2 mm/kg) and 7 mm long. The LA/Ao ratio was 2, and the end-diastolic left ventricle diameter Z score was +2.2. The normal blood flow in the descending aorta and its branches (coeliac trunk, renal arteries) during diastole was observed. Although the failed response to medical treatment was observed, the case was discussed in the JCC meeting, and we decided to perform transcatheter PDA closure using the APO (Abbott Structural Heart, Plymouth, MN, USA) from the pulmonary arterial end.

### 2.3. Case 3

The second child from the twin pregnancy, which was complicated by the intrauterine fetal demise at 20 GW, was born via emergency cesarian section at 25 GW (BW 870 g, BL 36 cm, AS 4/5). Initially, the patient was treated in a regional hospital. From delivery, she showed signs of respiratory distress, and she was intubated. The patient received surfactant endotracheal four times, but she was dependent on mechanical ventilation with high tidal volume for over a month. Initial antimicrobial therapy (ampicillin, amikacin) and caffeine-citrate were applied. Due to an acute pulmonary and intracranial hemorrhage (grade 2), tranexamic acid was added to the therapy. Meropenem and vancomycin were prescribed in the third DOL, along with fluconazole prophylactically. The convulsions were stopped with the use of phenobarbitone. Thrombocytopenia was registered in the third DOL.

The patient became hypotensive, so dual inotropic drugs were administered (dopamine and dobutamine). Due to hsPDA, paracetamol and ibuprofen were administered. However, in addition to the applied therapy, the hsPDA was maintained, and the patient became anuric, so a transfer to our Institute for further treatment was indicated.

Upon admission, the patient was hypotrophic, weighing 1322 g, and exhibited generalized swelling. The patient required mechanical ventilation and was anuric and hypotensive despite receiving inotropic support with dopamine and dobutamine, as well as diuretic treatment with furosemide and amiloride hydrochlorothiazide. Peritoneal dialysis was initiated on the second day of hospitalization. Due to progressive respiratory and circulatory deterioration, high-frequency oscillatory ventilation and dual inotropic drug support with epinephrine and norepinephrine were started. TTE examination revealed a widely open PDA, 2.8 mm in diameter on the pulmonary end (2.3 mm/kg) and 8 mm long. The LA/Ao ratio was 2.2, and the end-diastolic left ventricle diameter Z score was +3.6. The reversal of forward flow in the renal arteries during diastole was observed. Although the failed response to medical treatment was observed, the case was discussed in the JCC meeting, and we decided to perform transcatheter PDA closure using the APO (Abbott Structural Heart, Plymouth, MN, USA) from the pulmonary arterial end.

### 2.4. Inclusion Criteria

The inclusion criteria for our patients were as follows: a current weight greater than 700 g, a postnatal age of at least three days, clinical symptoms indicating a hsPDA (specifically, dependence on mechanical ventilation and prerenal acute kidney failure), failure of at least one or two courses of pharmacological treatment, and the presence of a significant PDA on echocardiography. Based on literature data, earlier closure of the PDA (within four weeks) is associated with better outcomes, such as quicker weaning from ventilatory support and improved growth. Therefore, we performed the intervention as early as possible (Table 1).

### 2.5. Intervention

**Figure 2:**
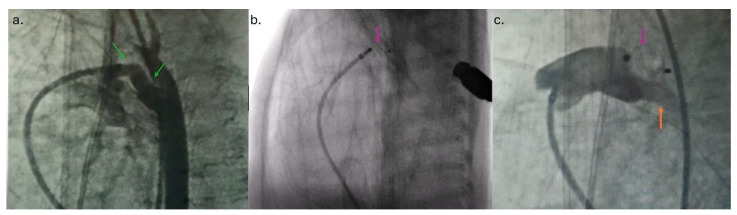
(**a**) Angiogram in the left lateral position indicated widely open ductus arteriosus (green arrow) and both pulmonary branches. (**b**) The entire Amplatzer Piccolo Occluder (pink arrow) was placed into ductus arteriosus. (**c**) Pulmonary artery angiogram after the device was released (orange arrow—left pulmonary artery).

The intervention was performed in a catheterization room under general anesthesia. The neonatal transport team transferred the patients to the Cath Lab. Both patients were on mechanical ventilation, so the position of the ETT was checked using fluoroscopy at the start of the procedure. To prevent oxygen-free radical damage to end organs, the goal oxygen saturation was 89–93% [7]. An air heater and plastic drape cover prevented hypothermia. A standard 4 Fr introducer sheath is placed in the right femoral vein using a 20-gauge needle, floppy-tipped 0.018 guide wire, and standard Seldinger technique using ultrasound guidance. The right Judkins catheter and 0.035″ Terumo guide wire were fed across the femoral vein, lower vena cava, right atrium, right ventricle, pulmonary artery, and PDA placed in the descending aorta. Across the right Judkins catheter, a 0.018′′ floppy tipped wire with a “hockey stick” curve was introduced in the aorta, and the right Judkins catheter was then exchanged for the Torqvue LP delivery catheter (Abbott, MN, USA) (0.046″). Biplane aortography [6] was performed in anteroposterior and left lateral planes [4]. The angiography (1.5 mL of contrast) revealed PDA Krichenco type E (Figure 2a) [8]. Before the aortography, a naso-gastric tube was filled with contrast to mark the aortic end of the PDA. In the first patient, the duct diameter on the pulmonary end was measured at 2.4 mm, and the length was 9 mm. In the second case, the pulmonary end diameter was 2.7 mm, and the length was 12 mm. We decided to use APO 9-PDAP-04-02-L and 9-PDAP-04-04-L, respectively. The entire device, with both retention discs, was implanted within the duct (Figure 2b). Before realizing the device, TTE pointed out adequate device position without descending aorta, left pulmonary branch obstruction, and a residual shunt (Figure 1c,d). Control angiography after the device was realized confirmed adequate APO position (Figure 2c). Prophylactic antibiotics and a heparin bolus (100 IU/kg) were administered. The intervention was uneventful; the fluoroscopy lasted 5 min, and we spent 3 mL of contrast. In the third case, epinephrine was continuously administered throughout the entire procedure.

### 2.6. Outcome

**Figure 3:**
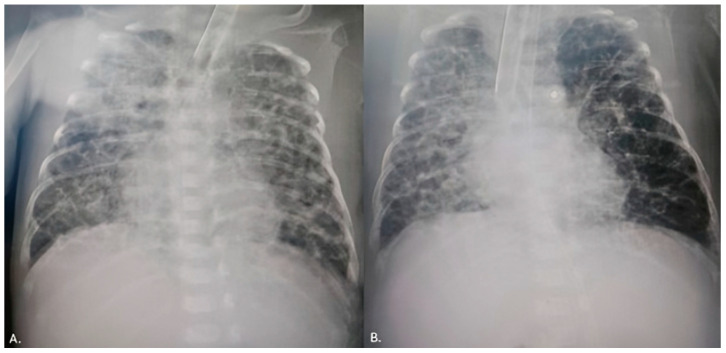
X-ray findings before (**A**) and after (**B**) transcatheter PDA closure.

The first patient was weaned from mechanical ventilation three days after the procedure. A control echocardiography examination revealed a decreased left ventricular EDD and LA/Ao ratio. Three weeks after the procedure, the patient was discharged with a body weight of 2300 g. The second patient was extubated 2 weeks after the procedure, and even an X-ray of the severe BPD displayed an improved condition (Figure 3). An echocardiography examination pointed out a decreased left ventricular EDD with mild pulmonary artery hypertension. Consequently, sildenafil was initiated. After 6 weeks, the patient was transferred to the regional hospital due to low body weight (1880 g). In the third case, the patient was placed on conventional mechanical ventilation immediately after the intervention, and the inotropes were discontinued after two days. Renal function was normalized. She weaned from mechanical ventilation after 3 weeks. 

## 3. Discussion

The hsPDA causes deterioration in the respiratory status of preterm infants by increasing ventilator support and oxygen requirement in previously wearable infants and heart failure development, and those patients require intervention [1,2]. Our pre-term newborns had hsPDA with an acute pulmonary hemorrhage and renal failure. Due to unsuccessful medical treatment, percutaneous closure was performed. Echocardiography revealed a widely open PDA leading to dilation of the left ventricle and left atrium. El Hajjar et al. suggest that when normalizing for body weight, a ductus diameter of 1.4 mm/kg or greater is associated with an increased risk of hypoperfusion. Other echocardiographic criteria used to assess hemodynamic significance include the reversal of forward flow in the descending aorta and its branches (coeliac trunk, renal arteries, cerebral arteries) during diastole and the dilation of the left atrium or left ventricle. The LA/Ao ratio is particularly sensitive when measured after the first day of life, with a value greater than 1.5 considered abnormal [8]. Two scoring systems have been developed to evaluate the significance of PDA: the El-Khuffash score, which correlates significantly with necrotizing enterocolitis (NEC), and the Shaare Zedek score, which correlates considerably with periventricular leukomalacia [9,10].

There is currently no consensus on the optimal timing for PDA closure in premature infants [5]. However, a study by Kikuchi et al. found that reduced cerebral blood flow caused by PDA in preterm infants negatively impacts long-term neurodevelopment. Timely interventions, including surgical treatment for PDA, should ideally be performed within the first five days after birth, which may significantly improve developmental outcomes for these infants [11]. On the other hand, very early (<72 h of age) or early (<7 to 14 days of age) routine treatment to induce PDA closure (regardless of hemodynamic significance) in preterm infants, either medically, via transcatheter, or surgically, can close the ductus but does not improve outcomes and is not recommended (level of evidence: 1A) [12].

Previously, after the unsuccessful drug treatment, surgical ligation was used to be performed, and until now, it has been our institutional protocol. However, clinical studies have shown that the use of the APO (Abbott Structural Heart, Plymouth, MN, USA), formerly known as the Amplatzer Duct Occluder II Additional Sizes (ADO II AS), is now FDA-approved for PDA closure in patients weighing 700 g or more [5]. Two other commonly used devices for transcatheter closure in premature infants are the Medtronic microvascular plug (MVP; Medtronic, Minneapolis, MN, United States) and the KA Medical Micro Plug (KA Medical, Saint Paul, MN, United States), and they are used off-label [13,14]. Consequently, we decided on APO. The device can be used to occlude a PDA that is 4 mm or less in diameter and 3 mm or more in length, based on intra-procedural echocardiograms or angiograms. Transcatheter closure (TCC) has gained popularity due to its significantly lower complication rate compared to surgical ligation (3.3% versus 25.7%) [3,5]. Nonetheless, TCC does carry potential intra-operative complications specific to the technique, including cardiac perforation (1.3%), vascular injury (1%), tricuspid valve injury (2–5%), and pulmonary artery and aortic obstruction (2%), which may occur due to device protrusion and embolization [15].

The techniques for PDA TCC vary based on the infant’s weight [4]. In children weighing less than 2 kg, femoral arterial access should be avoided due to the risk of ischemic injury to the leg and potential bleeding [5,7,11]. Additionally, a femoral artery diameter of less than 3 mm can lead to the loss of arterial pulse, even with ultrasound guidance during femoral artery access in pediatric patients, as young infants are particularly vulnerable to femoral injury [6]. To minimize the risk of arterial damage, we performed femoral vein punctures using ultrasound guidance in our patients.

The transthoracic echocardiogram (TTE) was used to determine the appropriate device size and monitor its deployment. To confirm the dimensions of the duct, we conducted a small-dose contrast angiogram (1 mL/kg). It is recommended that the device be placed intraductally to reduce the risk of protrusion into the aorta or the left pulmonary artery (LPA) and to prevent unintentional stenosis of these vessels caused by the device discs. For children weighing 2 kg or less, device size selection was based on achieving adequate disc compression while favouring a shorter device length of 2 mm. If there is any suspicion of obstruction in the aorta or LPA or significant residual ductal shunting, we would capture the device, reposition it, and potentially replace it with a different size [5]. The device was deployed under fluoroscopic and transesophageal echocardiogram (TEE) guidance. Post-procedure echocardiography showed unobstructed blood flow in the LPA and the aorta, with no residual flow noted.

Complete closure of PDA in our patients without any procedural complications was noticed. Namely, the APO was successfully implanted in 93–100% of patients from a cohort of 277 individuals receiving ADO II AS across 10 medical centres in Europe, including 99% of patients weighing ≤2 kg [5]. According to data from the IMPACT Registry Insights, 1549 patients (97.6%) < 2 kg had a successfully implanted device, major adverse events (MAE) occurred in 61 (3.8%), and the composite outcome occurred in 85 (5.3%) of the patients. The most common MAEs were device embolization requiring retrieval (1.3%), cardiac arrest (0.7%), cardiac tamponade (0.3%), device malposition or thrombus requiring surgery/intervention (0.6%), unplanned cardiac or vascular surgery 1.3%, arrhythmia requiring treatment (0.6%), and a major bleeding event (0.6%). Patients with the composite outcome, procedure failure, or MAE had a higher incidence of arterial access [13].

Our patients experienced clinical and echocardiographic improvement shortly after the intervention. Additionally, a post hoc analysis of the PDA randomized controlled trial cohort indicated that early elimination of the shunt could reduce respiratory morbidity and decrease the risk of pulmonary hypertension [13]. Given the lack of comparative data between conservative and definitive interventions in high-risk populations, it is crucial to understand the safety endpoints of percutaneous PDA closure in the care of increasingly smaller neonates.

We presented two cases of premature infants with hsPDA that were successfully closed percutaneously without complications. According to available data, these cases are the first of premature infants in Serbia and regional countries where hsPDA was closed percutaneously.

## 4. Conclusions

We performed uneventful percutaneous PDA closure in the two preterm newborns after unsuccessful medical treatment for the first time in our country. The patients had no complications; they weaned from mechanical ventilation after 3 and 10 days, respectively. Due to a significantly lower complication rate than surgical ligation, percutaneous PDA closure, we will strive to make percutaneous PDA occlusion a new standard for treatment in newborns, especially preterm newborns, in our country. Further research is needed to determine which PDAs require closure and the appropriate timing for closing the hsPDA in premature infants.

## Figures and Tables

**Table 1:** Summary of Three Cases.

	Case 1	Case 2	Case 3
gender	male	female	female
GW	29.6	26	25
BW (g)	1370	850	870
BL (cm)	39	36	36
AS	2/5	7	4/5
Clinical presentation	pulmonary hemorrhage hypotension AKI	broncho-pulmonary dysplasia hypotension	pulmonary hemorrhage intracranial hemorrhage circulatory insufficiency AKI
Echocardiography parameters - LA/Ao - EDD (mm) - EDD (Z score) - PDA (mm)	1.8 20 +2.89 2.36	2 14 +2.2 2.6	2.2 19 +3.6 2.8
INTERVENTION			
DOL	28	60	60
PDA diameter	2.3	2.5	2.8
Device	APO 9-PDAP-04-02-L	APO 9-PDAP-04-04-L	APO 9-PDAP-05-04-L
Inotropes	NO	YES	YES
Mechanical ventilation	3 days after	2 weeks after	3 weeks after
FOLLOW-UP			
Months	4	3	0.5
Echocardiography parameters - LA/Ao - EDD (mm) - EDD (Z score)	1.5 24 +1.8	1.2 13 0	1.3 17 +1.22

Abbreviations: GW—gestational week; BW—body weight; BL—body length; AS—Apgar score; AKI—acute kidney failure; LA—left atrium; Ao—aorta; EDD—end-diastolic diameter; PDA—patent ductus arteriosus; DOL—day of life; APO—Amplatzer Piccolo Occluder.

## Data Availability

The original contributions presented in this study are included in the article. Further inquiries can be directed to the corresponding author.

## Abbreviations

PDA—patent ductus arteriosus; GW—gestational week; BW—birth weight; AS—Apgar score; TTE—transthoracic echocardiography; LA—left atrium; Ao—aorta; APO—Amplatzer Piccolo Occluder; ADO II AS—Amplatzer Duct Occluder II Additional Sizes; BPD—bronchopulmonary dysplasia; DA—ductus arteriosus; PGE—prostaglandin E2; PGI2—prostacyclin; NO—nitric oxide; hsPDA—hemodynamically significant patent ductus arteriosus; NSAID—non-steroidal anti-inflammatory drugs; VLBW—very low birth weight; ELBW—extremely low birth weight; PROM—premature rupture of membranes; BL—body length; DOL—day of life; JCC—joint cardiology–cardiothoracic; ETT—endo tracheal tube; EDD—end-diastolic diameter; NEC—necrotizing enterocolitis; FDA—Food and Drug Agency; TCC—transcatheter closure; LPA—left pulmonary artery.
